# Adrenal Ganglioneuroma: Diagnosis, Presentation, and Management of a Rare Tumor

**DOI:** 10.7759/cureus.39977

**Published:** 2023-06-05

**Authors:** Maya M Eldin, Rachel E Daum, Pratima Kumar, John Uecker

**Affiliations:** 1 Department of Internal Medicine, The University of Texas at Austin Dell Medical School, Austin, USA; 2 Department of Endocrinology, The University of Texas at Austin Dell Medical School, Austin, USA; 3 Department of General Surgery, The University of Texas at Austin Dell Medical School, Austin, USA

**Keywords:** ganglioneuroma-retroperitoneal-imaging-pyelonephritis, hashimoto’s thyroiditis, adolescent and young adult (aya), histopathology examination, preventative care, adrenal glands, adrenal pheochromocytoma, adrenal ganglioneuroma, adrenalectomy

## Abstract

Adrenal ganglioneuromas are rare tumors arising from sympathetic ganglion cells that may present similarly to other adrenal tumors, making preoperative diagnosis challenging. We present a case of a young woman with a history of Hashimoto's thyroiditis who presented with hypertension and headaches. An abdominal CT scan revealed a large left adrenal mass, and while laboratory tests for catecholamines and metanephrines were normal, the suspicion for pheochromocytoma remained high given the size of the mass and persistent hypertension. The patient was started on alpha-blockers and beta-blockers in preparation for surgical removal. Pathology revealed a mature ganglioneuroma without evidence of malignancy, and postoperative blood pressure was normalized. We hypothesize that vessel compression from the large mass created functional stenosis, resulting in persistent hypertension. This case highlights the importance of a thorough workup for hypertension in young adults and routine preventative care visits to avoid delayed management. Adrenalectomy with histopathological examination remains the gold standard for treatment and diagnosis, and patients have a good prognosis following resection, with minimal need for recurrent therapy.

## Introduction

Ganglioneuromas are rare tumors arising from the neural crest cells in the sympathetic ganglion. They may occur in the adrenal glands, the posterior mediastinum, and the retroperitoneum [1-2]. However, presentation in the adrenal gland is rare and represents less than 5% of adrenal tumors. Though often asymptomatic, they may present similarly to neuroblastomas, pheochromocytomas, adrenal cortical adenomas, and adrenocortical carcinoma, making their preoperative diagnosis challenging [3]. Because of this, adrenalectomy with histopathological examination is the gold standard for treatment and diagnosis, and patients have a good prognosis following resection, with minimal need for recurrent therapy [4]. The rarity of this tumor may result in inaccurate diagnosis and delayed management, particularly in young adults with limited access to preventative care.

A unique problem presented itself in this patient’s case, as she first noted her elevated blood pressure two years ago but did not receive follow-up care in the interim. Due to her limited health insurance coverage, she was unable to establish care with a primary care physician and continued to monitor her blood pressure at home. This case highlights the need for yearly preventative care visits when working with young adult patients, particularly those who have a history of underutilizing services.

## Case presentation

A woman in her mid-20s presented to the emergency department for worsening hypertension and headaches. On arrival at the emergency room, her blood pressure was 200/120. The patient stated that she first noted elevated blood pressure two years ago while practicing blood pressure readings with classmates at nursing school. Since then, she had been periodically checking her blood pressure herself, as she had not established care with a primary care physician. The patient reported that, for the last two weeks, she had noticed a steady increase in her blood pressure, as well as an increased frequency of headaches, prompting her visit to the emergency department.

The patient denied any recent weight changes, palpitations, or shortness of breath. Her medical history was significant for Hashimoto’s thyroiditis, which was adequately treated with thyroid supplementation. She denied a family history of adrenal tumors, and physical examination did not show signs of Cushing syndrome.

**Figure 1:**
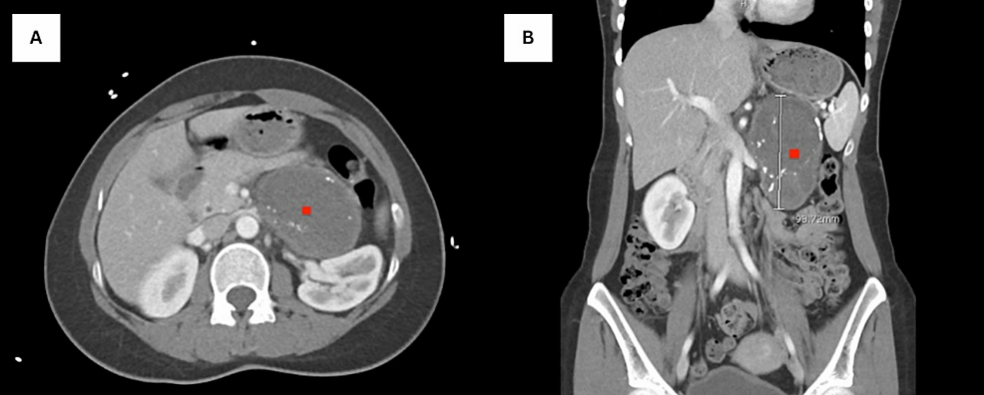
Computed tomography images (A) Clinical image showing axial computed tomography of well-defined, solid, encapsulated mass indicative of left adrenal ganglioneuroma (red square). (B) Coronal section of the left-sided adrenal ganglioneuroma around 98.72 mm in size (red square).

Laboratory evaluations, which included a complete metabolic panel, troponin level, urine pregnancy test, calcitonin, chromogranin, 8 AM cortisol and adrenocorticotropic hormone (ACTH), and 24-hour plasma-free metanephrines, were all within the normal range. The patient remained fasted overnight before collecting the samples. Given the patient’s young age and persistent hypertension, an abdominal computed tomography scan was obtained, which revealed a 10 cm complex cystic left adrenal mass with thick and thin internal septations and coarse calcifications (Figure 1A, 1B).

She was admitted to the intensive care unit for close blood pressure monitoring. Despite her non-diagnostic laboratory tests, the suspicion of a pheochromocytoma remained high, given the large size of the mass and the patient’s persistent hypertension. Because of this, she was started on alpha-blockers with doxazosin in the hospital. The patient was discharged home with a scheduled outpatient endocrinology follow-up to further optimize her blood pressure with beta-blockers.

**Figure 2:**
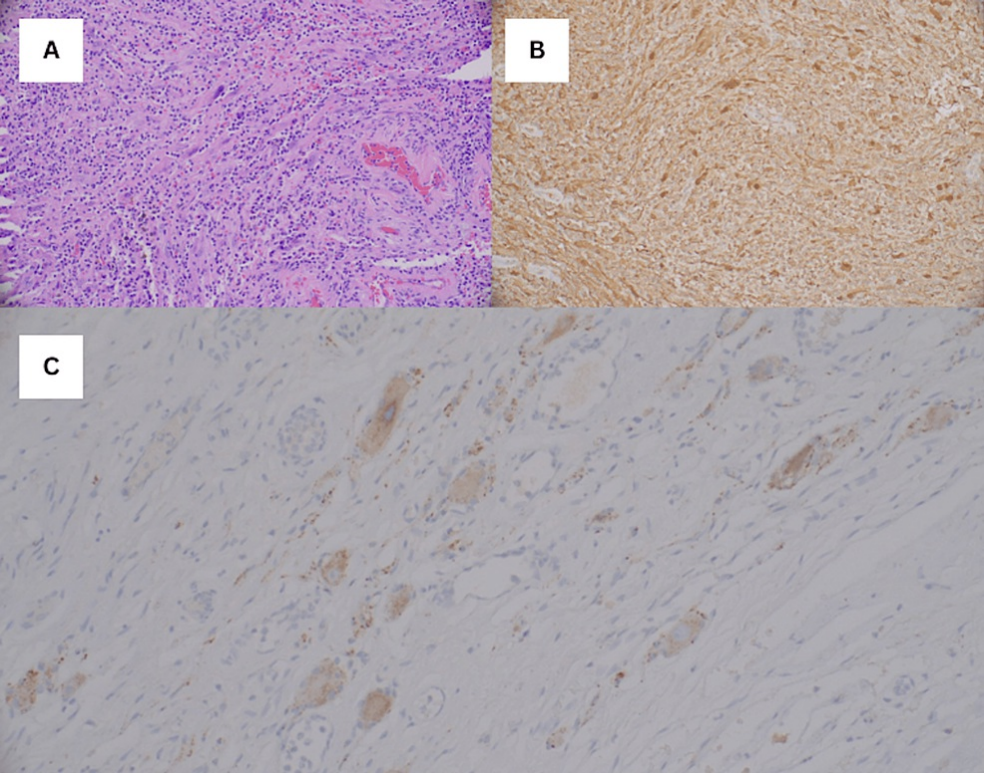
Immunohistochemistry features of the surgical specimen (A) Hematoxylin and eosin, (B) S-100 stain, and (C) Synaptophysin stain.

Although the above laboratory workup was relatively non-diagnostic, the large adrenal mass and persistent hypertension prompted the decision to proceed with surgical intervention with a robotic-assisted laparoscopic left adrenalectomy. The patient underwent general anesthesia with specific preparations in case of a potential pheochromocytoma. Medications such as alpha-adrenergic agonists and antagonists, beta-adrenergic antagonists, vasopressors, and vasodilators were prepared as part of the anesthesia management. During the procedure, thorough mobilization of the left colon and splenic flexure facilitated the visualization of the adrenal mass, followed by meticulous dissection, identification, clipping, and subsequent division of the adrenal vein. There were no complications during the surgery, and the patient was discharged the following day with outpatient endocrinology follow-up. Upon removal, the mass was fixed in formalin and sent for pathology interpretation. The surgical pathology was consistent with a mature ganglioneuroma with extensive ischemic degenerative changes with hyalinized fibrosis and dystrophic calcifications. There were patchy areas of spindle cell lesions with scattered ganglion cells and neurofilament-positive axons. The spindle cells were strongly positive for S100 stains, and the ganglion cells were positive for synaptophysin stains (Figure 2A-2C).

No malignancy was identified. Her blood pressure subsequently became normal leading to the cessation of the anti-hypertensive medications.

## Discussion

Ganglioneuromas are slow-growing, rare tumors that arise from the sympathetic ganglion cells in the neural crest and are commonly found in the mediastinum, retroperitoneum, and adrenal glands [1-2]. They have a higher prevalence in females than in males and are more likely to occur in adolescents with a median age at diagnosis of around seven years [5]. They are thought to arise sporadically, either from prior neuroblastoma maturation or spontaneously, as was seen in this patient, although associations with Turner syndrome and multiple endocrine neoplasia type 2 (MEN2) have been studied [6]. Although they are usually asymptomatic around their average size of 7 cm or below, they may cause a local mass effect on nearby organs, manifesting as abdominal pain, dyspnea, and cough. Adrenal ganglioneuromas make up only about 21% of ganglioneuromas diagnosed and can present similarly to neuroblastomas, pheochromocytomas, adrenal cortical adenoma, and adrenocortical carcinoma [3,7]. Histopathology of adrenal ganglioneuroma typically shows large, mature ganglion cells, Schwann cells, and perineural cells, with positive staining for neurofilaments and S100 on immunohistochemistry [7]. Surgical excision is the mainstay of treatment, although complete excision may not always be feasible due to capsule adherence to nearby structures and nerves. Autonomic dysfunction postoperatively is rare [5]. Lei et al. [8] conducted a retrospective study on 51 patients with adrenal ganglioneuroma and found a good prognosis and low recurrence rate after surgical treatment, highlighting the importance of surgical resection as the preferred treatment option. Long-term follow-up is recommended for monitoring [8].

Diagnosing adrenal ganglioneuromas can be challenging, particularly when compared to other adrenal masses. Although studies show ganglioneuroma can be suspected by the presence of punctate discrete calcifications, patients can have nonspecific findings on CT scans, including solid attenuation and calcifications as was found in this patient. In addition to a routine complete blood count and complete metabolic panel, patients with an incidental adrenal mass on a CT scan should receive a hormonal evaluation to assess for pheochromocytoma and primary aldosteronism. A diagnosis of ganglioneuroma can be suspected in patients who test negative for hormonal hypersecretion as these tumors are usually non-functional, although some studies found that around 37% of ganglioneuromas can secrete catecholamines [9]. Finally, the presence of vessel involvement and, if tested, a low, non-enhanced T1-weighted signal with gradual enhancement on dynamic MRI can also be indicative [10].

In the presented case, the patient’s persistent hypertension added to the diagnostic complexity. Hypertension in an otherwise young and healthy patient, especially in the setting of an adrenal mass, makes pheochromocytoma high on the differential diagnosis. Pheochromocytoma induces blood pressure elevation via the secretion of excess catecholamines, which also aid in its diagnosis. Plasma-free metanephrine (catecholamine breakdown products) levels provide the best test for pheochromocytoma (99% sensitivity; specificity 89%) [11]. However, failure to identify these masses can lead to life-threatening complications during surgery [12], highlighting the need for vigilant pre- and intraoperative blood pressure surveillance even in the setting of normal metanephrine levels. It is worth noting that Araujo et al. [13] (2022) reported three cases of composite pheochromocytoma-ganglioneuroma, which is a rare variant of adrenal ganglioneuroma characterized by the coexistence of a pheochromocytoma and a ganglioneuroma within the same tumor. Patients with composite pheochromocytoma-ganglioneuroma may present with symptoms related to excess catecholamine secretion, similar to those with pheochromocytomas [13]. In this case, since there were no histological findings of a pheochromocytoma, including no excess catecholamine secretion, positive staining for chromogranin A, or dopamine beta-hydroxylase, the presence of a composite pheochromocytoma-ganglioneuroma was effectively ruled out.

While hypertension is not a characteristic feature of ganglioneuroma, it is a reported sequela of large masses near the renal arteries. Multiple case reports have found both benign and malignant renal masses to be causes of medication-resistant hypertension [14-16]. Larger masses and those closer to the renal hilum were more likely to increase blood pressure, which corroborates the theory that hypertension may result from renal artery compression [17]. We postulate that renal artery compression was the cause of our patient’s hypertension, as her blood pressure normalized rapidly following mass excision. However, prior to her surgical pathology, pheochromocytoma was thought to be the cause of her hypertension; therefore, no specific imaging modalities, such as angiography or ultrasound, were performed to confirm this theory. 

Our patient case raises an important discussion on the role of preventative medicine. In this patient, she first noted an elevation in her blood pressure two years ago and continued to monitor it at home as she did not have an established primary care provider. Another barrier to her care was her limited insurance coverage. The importance of informed medical management including enhanced surveillance and prevention is highlighted in the young adult population. Young adults have been shown to utilize the healthcare system at lower rates compared with other groups, yet they utilize the emergency room at significantly higher rates compared with populations in cohorts younger and older than them [18]. In working with young adult patients, particularly those who have a history of underutilization of services, such as our patient, emphasis must be placed on annual preventative care visits.

## Conclusions

Adrenal ganglioneuromas are a rare but important differential diagnosis to consider in patients presenting with adrenal masses and secondary hypertension. The diagnostic workup and preoperative care should include a thorough evaluation, including imaging studies and biochemical tests, to determine the nature of the mass and rule out any associated endocrine abnormalities. The histological features of ganglioneuroma, such as the presence of mature ganglion cells and Schwann cells, should also be considered. Additionally, it is crucial to be aware of potential complications, such as renal artery compression causing medication-resistant hypertension, which can impact patient management and outcomes. By incorporating these considerations into the diagnostic and preoperative care of patients with adrenal ganglioneuromas, clinicians can improve patient outcomes and ensure appropriate management of this rare but important clinical entity.
